# Intra-articular corrective osteotomy for malunited Hoffa fracture: A case report

**DOI:** 10.1186/1758-2555-4-28

**Published:** 2012-08-07

**Authors:** Takao Iwai, Masayuki Hamada, Takahide Miyama, Konsei Shino

**Affiliations:** 1 Department of Orthopedic Sports Medicine, Hoshigaoka Koseinenkin Hospital, 4-8-1 Hoshigaoka Hirakata, Osaka 573-8511, Japan; 2 Faculty of Comprehensive Rehabilitation, Osaka Prefecture University, 3-7-30 Habikino, Habikino, Osaka, 583-8555, Japan; 3 Department of Orthopaedic Surgery, Hoshigaoka Koseinenkin Hospital, 4-8-1 Hoshigaoka, Hirakata City, Osaka, 573-8511, Japan

**Keywords:** Hoffa fracture, Malunion, Intra-articular corrective osteotomy

## Abstract

Hoffa fracture, an isolated coronal plane fracture of the posterior aspect of the femoral condyle, is known as an unstable, intra-articular fracture, and therefore, operative treatment is recommended. However, insufficient open reduction or failure of fixation may lead to malunion. We performed intra-articular corrective osteotomy for a malunited Hoffa fracture in a 31-year-old man and obtained good functional and radiographic results. This report suggests that intra-articular corrective osteotomy for malunited Hoffa fracture offers a good outcome and should be considered as salvage treatment.

## Background

Hoffa described isolated coronal plane fracture of the posterior aspect of the femoral condyle in 1904 [1]. The so-called Hoffa fracture is, by definition, an intra-articular fracture and has been reported to more commonly involve the lateral condyle [2]. Because this fracture is known as an unstable, intra-articular fracture, malunion is one of the late complications after nonoperative or even operative treatment. Malunions have been generally classified into extra-articular and intra-articular malunions. While corrective osteotomy for extra-articular malunions has been frequently reported, few reports describe the results of operative treatment for symptomatic intra-articular malunions. To the best of our knowledge, no reports have described salvage treatment for a malunited Hoffa fracture. Herein, we present our experience of intra-articular corrective osteotomy for a case of malunited coronal plane fracture.

### Case presentation

**Figure 1:**
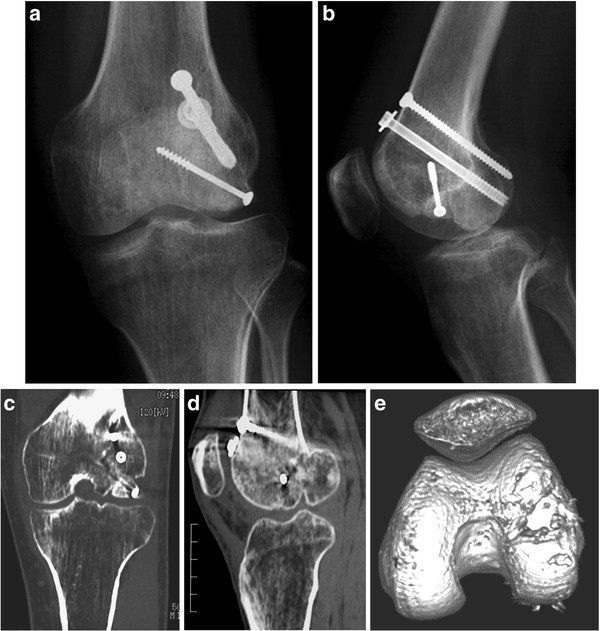
**Anteroposterior (a) and lateral (b) radiographs, and computed tomography scans (c-e) of the left knee 4 months after the first operation, showing the malunited lateral femoral condyle.** Figure 1**d** scale bar: 5 cm.

**Figure 2:**
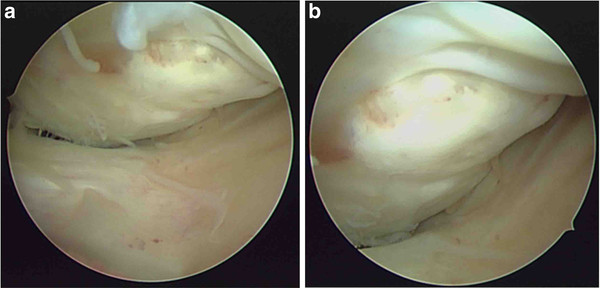
Diagnostic arthroscopy (a, b) showing severe comminution with depressed articular cartilage in the fractured area (25 × 20 mm.).

**Figure 3:**
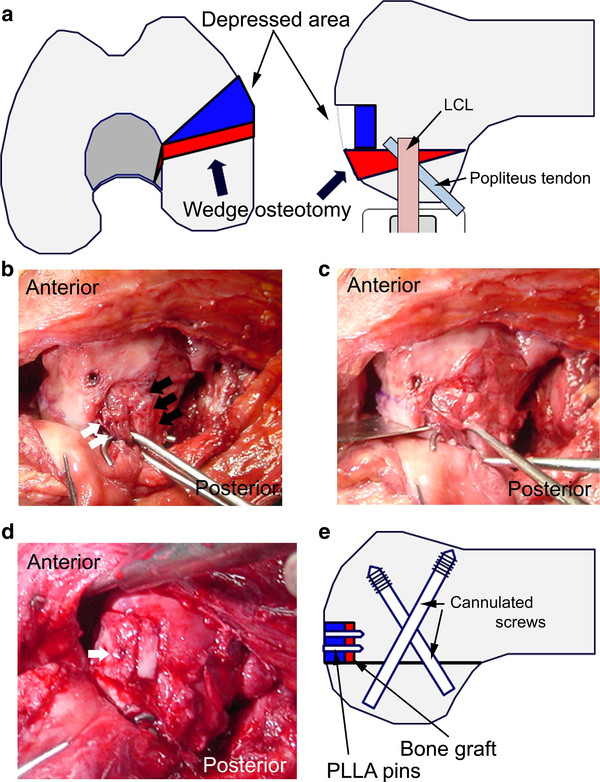
**a) Plan for 5-mm closed wedge osteotomy and elevation of the depressed portion. b**) Photograph of the lateral aspect of the left knee. The 2 skin hooks retracted the popliteus tendon (white arrow), and lateral collateral ligament (LCL) (black arrow). **c**) The skin hook retracted these structures to protect them from the bone saw during osteotomy. **d**) The depressed portion was elevated by using a bone graft and fixed using poly-l-lactic acid (PLLA) pins (white arrow). **e**) Schema after wedge osteotomy and internal fixation.

**Figure 4:**
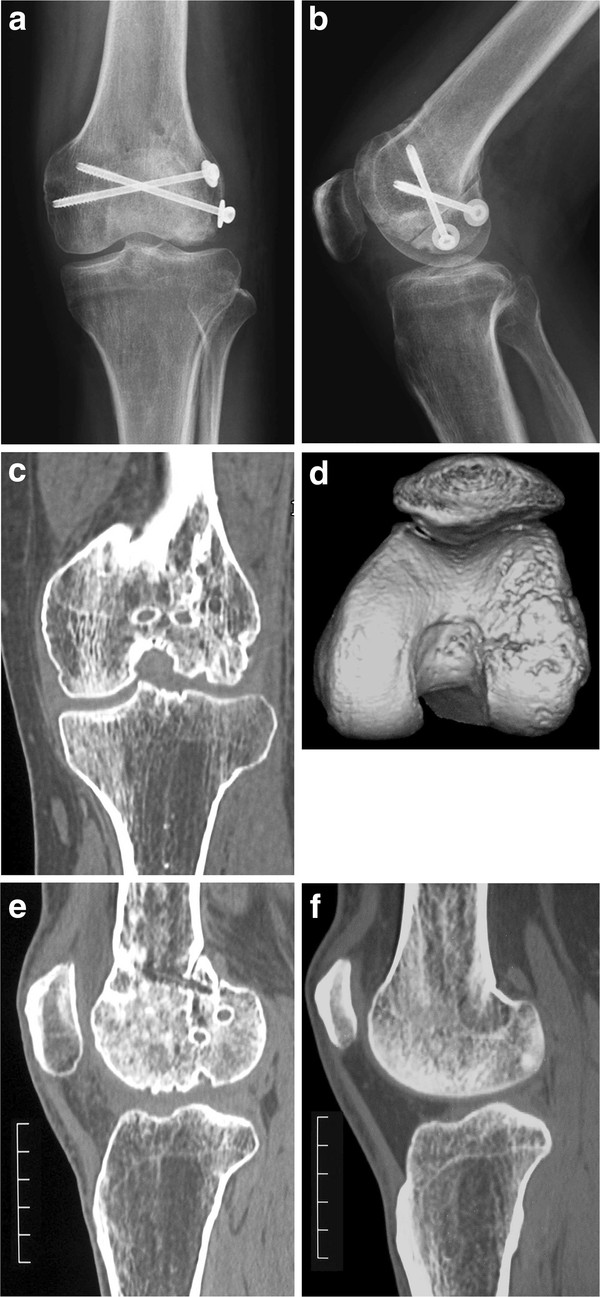
**Anteroposterior (a) and lateral (b) radiographs after corrective osteotomy. Computed tomography scans (c-e) at 1.5 years after corrective osteotomy shows complete bony union.** Computed tomography scan (**f**) of the contralateral side and f scale bar: 5 cm.

A 31-year-old man injured his left knee in a failed landing attempt during snowboarding. Subsequently, he was unable to bear weight. On the day of injury, he was diagnosed with a coronal plane fracture of the lateral femoral condyle and treated with open reduction and internal fixation using 3 screws at a different hospital. The fracture was type I according to the Letenneur classification [3]. At 2 months postoperatively, the range of motion was 0°/full extension to 40° of flexion, and manipulation of the knee joint was performed under anesthesia. At 4 months postoperatively, he was referred to our hospital for further treatment. On presentation at our hospital, his chief complaints were knee pain after walking and decreased range of knee flexion. The limitation of his knee flexion affected his daily life. He hoped to improve his knee flexion and knee pain in order to continue his job as a waiter. Physical examination showed that the range of motion was 0°/full extension to 120° of flexion, and the Lachman test was positive. No varus-valgus instability was noted. Plain radiographs and computed tomography scans revealed that the middle part of the lateral femoral condyle was depressed and the posterior part of the lateral femoral condyle was displaced posteriorly, although bony union was obtained (Figure 1). This incongruity of the lateral compartment of the knee joint was considered to be responsible for his complaints. At the second operation, performed 6 months after the first operation, diagnostic arthroscopy showed severe incongruity of his joint with depressed articular cartilage in the fractured area (25 × 20 mm) (Figure 2), with normal tibial surface, meniscus, and posterior cruciate ligament, and scarred anterior cruciate ligament (ACL). Exposure of the malunited fracture site was made with a lateral subvastus approach. With the knee flexed to 90°, the iliotibial band was retracted posteriorly, and the joint capsule was cut. This exposed the lateral collateral ligament (LCL), popliteus tendon, and the posterolateral corner. To reduce the malunited fracture, the malunited portion of the coronal fracture was correctly osteotomized (5-mm wedge osteotomy). Insertion of the LCL and popliteus tendon was preserved on the proximal fragment. The osteotomized fragment was fixed using two 5-mm cannulated screws. The depressed portion was elevated by using a bone graft from the osteotomized fragment, and was fixed using 2 poly-l-lactic acid (PLLA) pins (Figure 3). Plain radiographs taken in the operating room showed that the osteotomized fragment and depressed portion were reduced appropriately (Figures 4a, b). Mobilization was started on postoperative day 7. Partial weight-bearing was allowed from 2 months postoperatively, with full weight-bearing from 3 months postoperatively.

One year after the operation, knee pain was relieved and flexion of the joint had improved to 145°. Plain radiographs and computed tomography scans showed complete bony union (Figures 4c-e). Though we recommended reconstruction of the ACL at this point, the patient did not want to undergo this procedure because he had no complaint about the stability of his knee.

## Discussion

Hoffa fracture is an intra-articular fracture of the posterior aspect of the femoral condyle. In positions of knee flexion beyond 90°, the lateral femoral condyle is the leading part of the knee receiving oblique or lateral impacts [2]. Fractures result from direct trauma to this area, possibly with an element of abduction [2]. In this case, ACL injury and Hoffa fracture occurred at the same time. However, there is a big difference between the etiology of ACL injury and that of Hoffa fracture. To the best of our knowledge, no reports have described ACL injury associated with Hoffa fracture. We speculate that the mechanism of injury in this case was as follows: this patient was in the knee-in position when he landed during snowboarding. At that time, his ACL was injured, and his lateral femoral condyle was depressed because it received a high impact force. Then, he fell and bruised his knee while it was in a position of flexion of more than 90°; therefore, his knee received an axial loading force to the femoral condyle resulting in a Hoffa fracture.

Surgical fixation is the recommended method of treatment for Hoffa fractures [2-5]. Internal fixation of unicondylar fractures allows stable reconstruction of the distal articular surface of the femur and permits early postoperative motion of the knee [5]. Conversely, nonoperative management often leads to malunion or nonunion of the fracture. In this case, surgical fixation was performed at a local hospital, but open reduction proved insufficient. When the patient was referred to our hospital, his knee showed 2 major problems. We speculated that these problems caused his complaint. First, the posterior part of the lateral femoral condyle was posteriorly displaced. During deep flexion kneeling, the posterior surface of the tibia and posterior aspect of the femoral condyle are in direct contact [6]. Thus, a malunited femoral condyle may prevent hyperflexion. Second, the middle part of the lateral femoral condyle was depressed. Cartilage depression causes knee pain and contributes to the development of future posttraumatic osteoarthritis.

Though no reports have described corrective osteotomy for malunited Hoffa fractures, corrective osteotomy is reportedly effective for malunited intra-articular fractures of the proximal tibia and distal radius [7,8]. In addition, Kerkhoffs et al. reported that articular cartilage elevation using a bone graft was effective for depression of the articular cartilage and good functional results were obtained [8]. With reference to these reports, we used a combined method (elevation of the articular cartilage and intra-articular corrective osteotomy) in order to achieve a more anatomical reconstruction. Complete bony union was obtained at the osteotomized site; knee pain was relieved and joint flexion improved to 145°. In this case, the length of the osteotomized fragment was only 5 mm, but his knee flexion has improved. This result showed that even minimal malunion may cause decreased range of knee flexion. We believe that primary accurate operative treatment is very important for preventing malunion of Hoffa fractures. However, if malunion at the fracture site occurs after the first operation, intra-articular corrective osteotomy should be considered as salvage treatment.

## Consent

Written informed consent was obtained from the patient for publication of this case report and the accompanying images. A copy of the signed written consent form is available for review by the Editor-in-Chief of this journal.

## Competing interests

The authors declare that they have no competing interests.

## Authors’ contributions

TM and MH carried out the surgical treatment, TI and MH discussed the results, and KS advised on the treatment plan. All authors have read and approved the final manuscript.

## Author details

^1^Department of Orthopedic Sports Medicine, Hoshigaoka Koseinenkin Hospital, 4-8-1 Hoshigaoka Hirakata, Osaka 573-8511, Japan. ^2^Faculty of Comprehensive Rehabilitation, Osaka Prefecture University, 3-7-30 Habikino, Habikino, Osaka 583-8555, Japan. 3Department of Orthopaedic Surgery, Hoshigaoka Koseinenkin Hospital, 4-8-1 Hoshigaoka, Hirakata City, Osaka 573-8511, Japan.
